# Vascular Protective Role of Samul-Tang in HUVECs: Involvement of Nrf2/HO-1 and NO

**DOI:** 10.1155/2016/9580234

**Published:** 2016-06-05

**Authors:** Eun Sik Choi, Yun Jung Lee, Chang Seob Seo, Jung Joo Yoon, Byung Hyuk Han, Min Cheol Park, Dae Gill Kang, Ho Sub Lee

**Affiliations:** ^1^College of Oriental Medicine and Professional Graduate School of Oriental Medicine, Wonkwang University, 460 Iksandae-ro, Iksan, Jeonbuk 54538, Republic of Korea; ^2^Hanbang Body-Fluid Research Center, Wonkwang University, 460 Iksandae-ro, Iksan, Jeonbuk 54538, Republic of Korea; ^3^K-Herb Research Center, Korea Institute of Oriental Medicine, 1672 Yuseong-daero, Yuseong-gu, Daejeon 34054, Republic of Korea; ^4^Department of Oriental Medical Ophthalmology & Otolaryngology & Dermatology, College of Oriental Medicine, Wonkwang University, 460 Iksandae-ro, Iksan, Jeonbuk 54538, Republic of Korea

## Abstract

Samul-Tang (Si-Wu-Tang, SMT), composed of four medicinal herbs, is a well-known herbal formula treating hematological disorder or gynecologic disease. However, vascular protective effects of SMT and its molecular mechanisms on the vascular endothelium, known as the central spot of vascular inflammatory process, are not reported. The aim of this study was to investigate vascular protective effects of SMT water extract in human umbilical vein endothelial cells (HUVECs). Water extract of SMT was prepared and identified by HPLC-PDA analysis. Expression of cell adhesion molecules (CAMs) and heme oxygenase-1 (HO-1) and translocation of nuclear factor-kappa B (NF-*κ*B) and nuclear factor-erythroid 2-related factor 2 (Nrf2) were determined by western blot. Nuclear localization of NF-*κ*B and Nrf2 was visualized by immunofluorescence and DNA binding activity of NF-*κ*B was measured. ROS production, HL-60 monocyte adhesion, and intracellular nitric oxide (NO) were also measured using a fluorescent indicator. SMT suppressed NF-*κ*B translocation and activation as well as expression of CAMs, monocyte adhesion, and ROS production induced by TNF-*α* in HUVECs. SMT treated HUVECs showed upregulation of HO-1 and NO which are responsible for vascular protective action. Our study suggests that SMT, a traditionally used herbal formula, protects the vascular endothelium from inflammation and might be used as a promising vascular protective drug.

## 1. Introduction

Patients with vascular dysfunction are more likely to develop several complications like hypertension, congestive heart failure, angina pectoris, thrombosis, and atherosclerosis and these are pathologically related to each other. Recent studies suggest that the vascular endothelium, the inner lumen of blood vessels, is emphasized as the central spot of vascular inflammatory process [1]. The endothelium regulates vascular tone, proliferation, and permeability of inflammatory inducers or infiltration of leukocytes [2]. For inflammatory cascade to occur, selectins and CAMs (cellular adhesion molecules) are required which are expressed by inflammatory cytokines such as TNF-*α* [3] and ROS/NF-*κ*B pathway plays as a key mediator [4, 5]. TNF-*α* increases production of ROS (reactive oxygen species), stimulating redox signaling pathways resulting in atherogenesis [6], and stimulates NF-*κ*B (nuclear factor-kappa B), a transcription factor mediating the expression of inflammatory genes such as CAMs [7].

Conversely, some genes including HO-1 (heme oxygenase-1) are involved in vascular protection against inflammatory process. HO-1 is an enzyme that catalyzes degradation of heme to ferric iron, CO (carbon monoxide), and biliverdin [8], which is converted to bilirubin by biliverdin reductase [9]. Metabolites (ferric iron, CO, and bilirubin) produced by HO-1 are known to have antioxidative, anti-inflammatory, and antiatherogenic effects [10, 11]. In addition, HO-1 expression can also suppress atherosclerosis resulting from environmental factors such as smoking and air pollution [12]. NO (nitric oxide) is known as a vasodilator that can relax smooth muscle but also exerts antiatherogenic actions to inhibit adhesion of leukocyte and platelet on the endothelium [13].

Samul-Tang (SMT), also known as Si-Wu-Tang or the four-agent decoction, is a well-known herbal prescription traditionally used to treat women's illnesses such as anemia [14], dysmenorrhea [15, 16], and postpartum weakness resulting from hematological disorders defined as blood deficiency and blood stasis in traditional Korean medicine. SMT is recorded in several formularies including* Treasured Mirror of Eastern Medicine* (*Donguibogam*) and consists of 4 medical herbs: Angelicae Gigantis Radix (*Angelica gigas *Nakai, root), Cnidii Rhizoma (*Ligusticum officinale* Makino, rhizome), Rehmanniae Radix Preparata (*Rehmannia glutinosa* Gaertn. DC., rhizome, steamed and dried), and Paeoniae Radix (*Paeonia lactiflora* Pall., root). Recently, pharmacological studies were performed with SMT and it was proven to exert hematopoietic [17, 18], antipruritic [19], and antidermatitis [20] effects. To our knowledge, though tonifying effects of SMT are well known [21], vascular protective effects of SMT and its molecular mechanisms are not reported yet. Here, we report effects of SMT water extracts as a complementary or alternative therapeutic drug on vascular inflammation in human umbilical vein endothelial cells (HUVECs).

## 2. Materials and Methods

### 2.1. Plant Materials

The four crude herbs forming SMT were purchased from Omniherb (Yeongcheon, Korea) in February 2008. The origin of each herbal medicine was taxonomically identified by Professor Je Hyun Lee, Dongguk University, Gyeongju, Korea. A voucher specimen (2008-KE25-1~KE25-4) has been deposited at the K-Herb Research Center, Korea Institute of Oriental Medicine.

### 2.2. Chemicals and Reagents

Ferulic acid and 5-hydroxymethyl-2-furaldehyde (5-HMF) were purchased from Sigma-Aldrich (St. Louis, MO, USA). Albiflorin and paeoniflorin were the products of Wako (Osaka, Japan). Nodakenin was purchased from NPC BioTechnology Inc. (Daejeon, Korea). The purity of all reference standards was ≥98.0%. HPLC-grade methanol, acetonitrile, and water were obtained from J.T.Baker (Phillipsburg, NJ, USA). Glacial acetic acid, analytical reagent grade, was purchased from Junsei (Tokyo, Japan). RPMI 1640, fetal bovine serum, TNF-*α*, tissue culture reagents, 2′,7′-bis(2-carboxyethyl)-5(6)-carboxyfluorescein acetoxy-methylester (BCECF-AM), DAF-FM diacetate, and CM-H_2_DCFDA, Alexa Fluor 488 and 594 conjugated second antibodies were purchased from Invitrogen (San Diego, CA). Biotin 3′ End DNA Labeling Kit, LightShift® Chemiluminescent EMSA Kit, Biodyne® Precut Nylon Membranes, Lipofectamine LTX reagent, and* Renilla*-Firefly Luciferase Dual Assay Kit were purchased from Pierce Biotechnology (Rockford, USA). Primary antibodies, including mouse anti-ICAM-1, goat anti-VCAM-1, rabbit anti-E-selectin, mouse anti-NF-*κ*B, mouse anti-p-I*κ*B-*α*, rabbit anti-HO-1, and rabbit anti-Nrf2, were purchased from Santa Cruz Biotechnology (CA, USA). Donkey anti-goat IgG-H+I were purchased from Bethyl (Montgomery, USA) and goat anti-rabbit IgG and goat anti-mouse IgG were purchased from Enzo (Farmingdale, USA).

### 2.3. Preparation of SMT Decoction

SMT extract was deposited at the Herbarium of the K-Herb Research Center, Korea Institute of Oriental Medicine (Daejeon, Korea). SMT (18.76 g) is composed of four herbs, Angelica Gigantis Radix (*Angelica gigas *Nakai, root, 4.69 g), Cnidii Rhizoma (*Ligusticum officinale* Makino, rhizome, 4.69 g), Rehmanniae Radix Preparata (*Rehmannia glutinosa* (Gaertn.) DC., rhizome, steamed and dried, 4.69 g), and Paeoniae Radix (*Paeonia lactiflora* Pall., root, 4.69 g). Totally, 2.0 kg of SMT was extracted in distilled water at 100°C for 2 h using an electric extractor (COSMOS-660; Kyungseo Machine Co., Incheon, Korea). The solution was filtered using a standard sieve (number 270, 53 *μ*m; Chung Gye Sang Gong Sa, Seoul, Korea), evaporated to dryness at 40°C under vacuum (Eyela N-11, Tokyo, Japan), and freeze-dried (PVTFD10RS, ilShinBioBase, Yangju, Korea) and retained at −70°C until required. The amount of water extract was 667.3 g (yield: 33.3%).

### 2.4. High-Performance Liquid Chromatography (HPLC) Analysis of SMT

The chromatographic analysis was performed using the Shimadzu Prominence LC-20A series (Shimadzu Co., Kyoto, Japan) consisting of a solvent delivery unit (LC-20AT), online degasser (DGU-20A_3_), column oven (CTO-20A), autosample injector (SIL-20AC), and photodiode array detector (PDA, SPD-M20A). The data were acquired and processed by LCsolution software (Version 1.24). All analytes were separated on a Phenomenex Gemini C18 (250 × 4.6 mm, 5 *μ*m, Torrance, CA, USA) and maintained at 40°C. The mobile phases consisted of 1.0% (v/v) aqueous acetic acid (A) and 1.0% (v/v) acetic acid in acetonitrile (B). The gradient flow was as follows: 5–60% B for 0–40 min, 60–100% B for 40–45 min, 100% B for 45–50 min, and 100–5% B for 50–55 min. The flow-rate was kept 1.0 mL/min and injection volume was 10 *μ*L. The analytes were detected at 230 nm for albiflorin and paeoniflorin, 280 nm for 5-HMF, 320 nm for ferulic acid, and 330 nm for nodakenin. For quantitative analysis, lyophilized SMT extract (200 mg) was dissolved in 20 mL of distilled water and then the solution was filtered through a 0.2 *μ*m GHP syringe filter (SmartPor, PALL Life Sciences, Ann Arbor, MI, USA) before HPLC injection.

### 2.5. Cell Cultures

Human umbilical vein endothelial cells (HUVECs) and HL-60, human promyelocytic leukemia cell line, were purchased from the American Type Culture Collection (ATCC, Manassas, VA). Cells were cultured with RPMI 1640 containing 10% fetal bovine serum and penicillin-streptomycin and maintained in a humidified incubator containing 5% CO_2_ at 37°C.

### 2.6. Western Blot Analysis

Cell homogenates were separated on 10% SDS-polyacrylamide gel electrophoresis and transferred to nitrocellulose paper. Blots were then washed with H_2_O, blocked with 5% skimmed milk powder in tris-buffered saline Tween-20 (TBS-T) (10 mM tris-HCl, pH 7.6, 150 mM NaCl, and 0.05% Tween-20) for 1 h, and incubated with the appropriate primary antibody at dilutions recommended by the supplier. Then the membrane was washed, and primary antibodies were detected with secondary antibodies conjugated to horseradish peroxidase, and the bands were visualized with enhanced chemiluminescence (Amersham Bioscience, Buckinghamshire, UK). Protein expression levels were determined by analyzing the signals captured on the nitrocellulose membranes using the ChemiDoc image analyzer (Bio-Rad Laboratories, Hercules, CA).

### 2.7. Preparation of Cytoplasmic and Nucleus Extracts

The cells were scraped in cold PBS on ice and centrifuged at 13,000 rpm for 10 min at 4°C. Nuclear and cytoplasmic extracts were extracted with NE-PER Nuclear and Cytoplasmic Extraction Reagents (Pierce Biotechnology). After cytosolic protein was extracted with cytoplasmic extraction reagents I and II, nuclear pellet was then resuspended with 100 *μ*L of nuclear extraction reagent. Nuclear protein extracts were immediately transferred to a clean prechilled tube and all extracts were stored at −80°C until use.

### 2.8. Monocyte-HUVEC Adhesion Assay

In adhesion assay, 1.2 × 10^6^ of HUVECs were seeded in 24-well plates. HUVECs were grown to confluence in 24-well culture plates, pretreated with SMT for 30 min, and stimulated with TNF-*α* for 6 h. Then the HL-60 cells were labeled with 10 *μ*M BCECF-AM for 1 h at 37°C and washed twice with growth medium. This was followed by adding 2.5 × 10^5^ of the labeled HL-60 cells to the HUVEC and incubating them in a CO_2_ incubator for 1 h. The nonadherent HL-60 cells were removed from the plate by washing with PBS, and the HL-60 cells bound to the HUVEC were measured by fluorescence microscopy and then lysed with 50 mM tris-HCI, pH 8.0, containing 0.1% SDS. The fluorescent intensity was measured using a spectrofluorometer (Infinite F200 PRO, TECAN) at excitation and emission wavelengths of 485 and 535 nm, respectively.

### 2.9. Intracellular ROS Production Assay

The fluorescent probe, CMH_2_DCFDA, was used to determine the intracellular generation of ROS. Briefly, the confluent HUVECs in the 24-well culture plates were pretreated with SMT for 30 min. After removing from the wells, the HUVECs were incubated with 20 *μ*M CM-H_2_DCFDA for 6 h and then stimulated with TNF-*α*. The fluorescence intensity was measured by spectrofluorometer (Infinite F200 PRO, TECAN) and examined under a fluorescence microscope (Eclipse Ti, Nikon).

### 2.10. Intracellular NO and Nitrite Production Assay

The fluorescent probe, DAF-FM diacetate, was used to determine the intracellular generation of NO. The confluent HUVECs in the 6-well culture plates were pretreated with DAF-FM for 1 h. After removing excess probe from the wells, the HUVECs were treated with SMT for 30 min. The fluorescence intensity was measured by a spectrofluorometer (Infinite F200 PRO, TECAN) and examined under a fluorescence microscope (Eclipse Ti, Nikon). Nitrites were measured with 50 *μ*L of cell cultured medium, Griess assay solution, 50 *μ*L of 1% solution of sulfanilamide diluted in 5% phosphoric acid, 50 *μ*L of 0.1% N-1-napthylethylenediamine dihydrochloride (NED) diluted in sterile water. Sodium nitrite (1–100 *μ*M) was used to set standard curve. Absorbance was read at 540 nm using a spectrometer (Infinite F200 PRO, TECAN).

### 2.11. Electrophoretic Mobility Shift Assay (EMSA)

EMSA for NF-*κ*B was performed in the nuclear fraction using LightShift Chemiluminescent EMSA Kit (Pierce Biotechnology, Rockford, IL) following the manufacturer's protocol. Briefly, DNA was biotin-labeled using the biotin 3′ end-labeling kit (Pierce Biotechnology), ds NF-*κ*B oligonucleotide (5′-AGTTGAGGGGACTTTCCCAGGC-3′ and 3′-TCAACTCCCCTGAAAGGGTCCG-5′) incubated in a tube with terminal deoxynucleotidyl transferase (TdT) buffer, and ultrapure water at 37°C for 30 minutes. To extract labeled DNA, chloroform : isoamyl alcohol (24 : 1) was added and centrifuged at 13,000 rpm. The top aqueous phase containing the labeled DNA was further used and each binding reaction contained 1x binding buffer (100 mM tris, 500 mM KCl, and 10 mM dithiothreitol, pH 7.5), 2.5% glycerol, 5 mM MgCl_2_, 50 ng/mL poly(dI–dC), 0.05% NP-40, 2.5 mg of nuclear extract, and 20 to 50 fmol of biotin end-labeled target DNA. The contents were incubated at room temperature for 20 minutes. To this reaction mixture, loading buffer was added, subjected to gel electrophoresis on a native polyacrylamide gel, and transferred to a nylon membrane. After transfer was completed, DNA was cross-linked to the membrane at 120 mJ/cm^2^ using a UV cross-linker equipped with 254 nm bulb. The biotin end-labeled DNA was detected using streptavidin-HRP conjugate and a chemiluminescent substrate. The membrane was developed using ChemiDoc (Bio-Rad).

### 2.12. Luciferase Promoter Assay

Sixty to seventy percent confluent cells were transiently cotransfected with the plasmids using Lipofectamine LTX (Invitrogen, Carlsbad, CA) according to the manufacturer's protocol. Briefly, transfection mixture containing 5 *μ*g of the pGL3-NF-*κ*B-Luc or* Renilla* and 5 *μ*L of media was mixed with the Lipofectamine LTX reagent and added to the cells. After 48 h, the cells were treated with SMT for 30 min and stimulated with TNF-*α* for 6 h and then lysed. The luciferase activities were determined using* Renilla*-Firefly Luciferase Dual Assay Kit (Thermo Scientific, Rockford, IL). The luciferase assay activity was normalized with respect to the* Renilla* activity and was expressed as a percentage of the activity of the control.

### 2.13. Immunofluorescence Microscopy

For localization of NF-*κ*B and Nrf2, HUVECs were grown on cover glass and treated as described in all figures' captions. Cells were then fixed in 1% formaldehyde and permeabilized with 0.1% Triton X-100. The cells were probed with NF-*κ*B or Nrf2 antibody followed by Alexa Fluor 488 or 594 conjugated secondary antibody, respectively. To visualize the nuclei, cells were then treated with 1 *μ*g/mL of DAPI for 10 min. Cells were finally washed three times with PBS, and coverslips were mounted with mounting solution onto glass slides and examined under a fluorescence microscope (Eclipse Ti, Nikon).

### 2.14. Statistical Analysis

All the experiments were repeated at least three times. The results were expressed as a mean ± SE, and the data were analyzed using one-way ANOVA followed by Student's *t*-test to determine any significant differences. *p* < 0.05 was considered as statistically significant.

## 3. Results

### 3.1. HPLC Analysis of SMT

**Figure 1:**
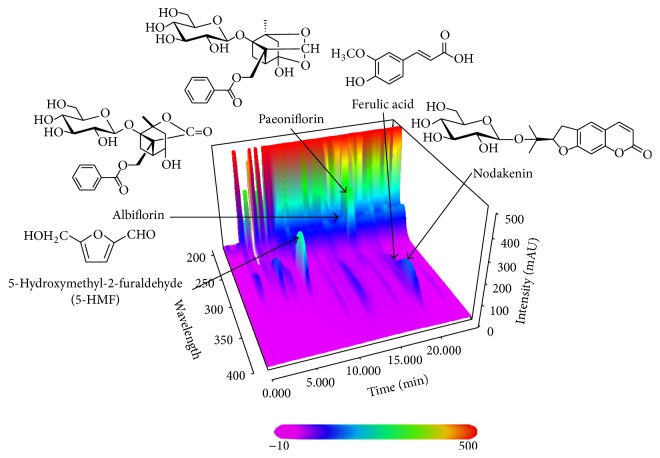
Three-dimensional chromatogram of Samul-Tang by HPLC-PDA.

The developed HPLC-PDA method was subsequently applied for the quantitative analysis of the five marker compounds in SMT. Consequently, five compounds were eluted within 30 min and typical three-dimensional chromatogram using HPLC-PDA detector is shown in Figure 1. The retention times of the five marker components, 5-HMF, albiflorin, paeoniflorin, ferulic acid, and nodakenin, were 8.07, 16.05, 17.00, 19.65, and 20.15 min, respectively. The correlation coefficient (*r* ^2^) of the five compounds showed good linearity as ≥0.9999. Using optimized chromatography conditions, the amounts of the five compounds, 5-HMF, albiflorin, paeoniflorin, ferulic acid, and nodakenin, in SMT were 2.79 ± 0.04, 2.91 ± 0.06, 15.18 ± 0.12, 1.08 ± 0.02, and 5.99 ± 0.10 mg/g, respectively.

### 3.2. Effect of SMT on TNF-*α* Induced Expression of Cell Adhesion Molecules in HUVECs

**Figure 2:**
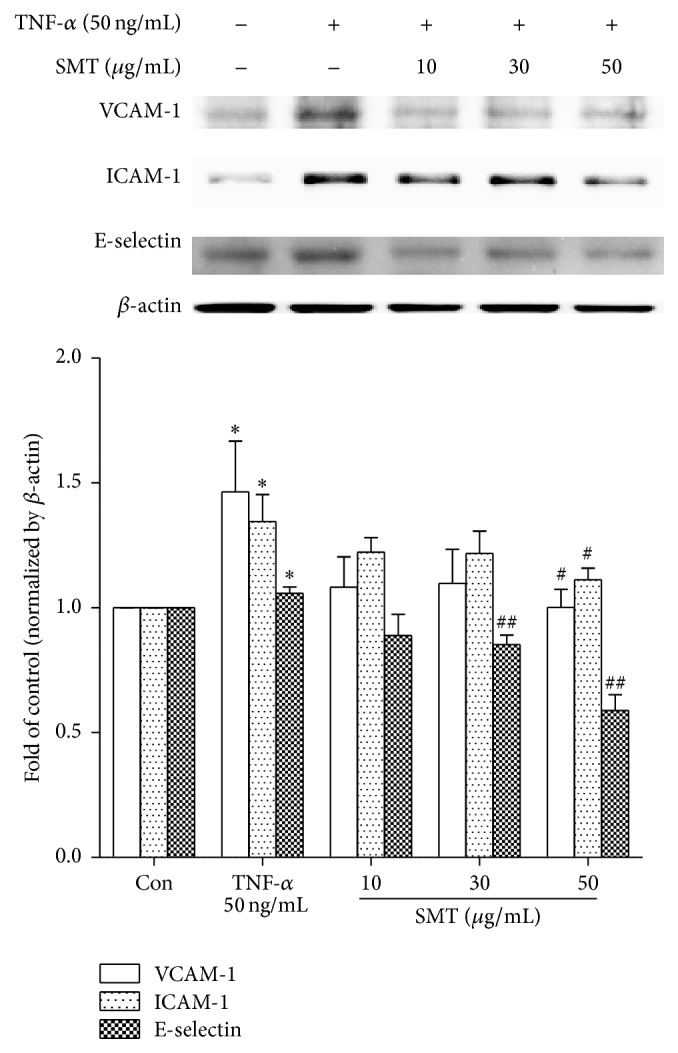
Effects of SMT on TNF-*α* induced cell adhesion molecules expression in HUVECs. Cells were treated with TNF-*α* (50 ng/mL) for 6 h in the absence or pretreatment of SMT (10, 30, and 50 *μ*g/mL) for 30 min. Bar represents the mean ± SEM of 3 independent experiments. ^*∗*^ *p* < 0.05 versus con group. ^#^ *p* < 0.05 and ^##^ *p* < 0.01 versus TNF-*α* group.

MTT assay was performed to investigate cytotoxic potential of SMT on HUVECs. Cells were treated with different concentrations of SMT (10–200 *μ*g/mL) for 24 h and performed as described in Section 2. Cell viability of HUVECs was not influenced by treatment of SMT alone in all concentrations ranging from 10 to 200 *μ*g/mL concentration. On the basis of this result, concentration of SMT was less than 200 *μ*g/mL in following experiments (data not shown). To investigate the effects of SMT on expression of cell adhesion molecules (CAMs) such as ICAM-1 (intracellular adhesion molecule-1), VCAM-1 (vascular cell adhesion molecule-1), and E-selectin (endothelial-selectin) induced by TNF-*α* in HUVECs, western blot was performed. As shown in Figure 2, 6 h of induction with TNF-*α* (50 ng/mL) significantly upregulated protein expression of VCAM-1, ICAM-1, and E-selectin compared to the control group (^*∗*^ *p* < 0.05), whereas SMT pretreatment for 30 min inhibited VCAM, ICAM, and E-selectin expression against TNF-*α* induction over 30 *μ*g/mL (^#^ *p* < 0.05, ^##^ *p* < 0.01).

### 3.3. Effect of SMT on TNF-*α* Induced Monocyte Adhesion in HUVECs

**Figure 3:**
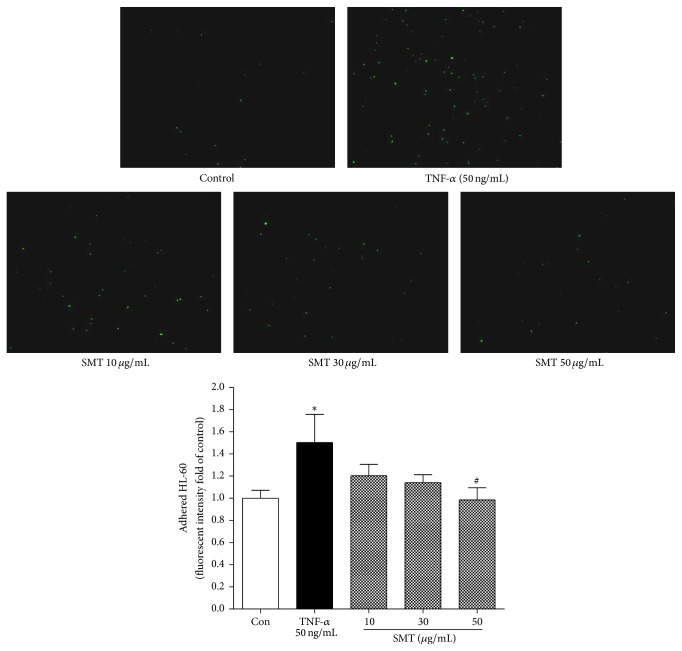
Effects of SMT on TNF-*α* induced cell adhesion of HL-60 in HUVECs. HUVECs were treated with TNF-*α* (50 ng/mL) for 6 h in the absence or pretreatment of SMT (10, 30, and 50 *μ*g/mL) for 30 min and then incubated with BCECF-AM labeled HL-60 cells. Adhered monocytes were captured with fluorescent microscope. Bar represents the mean ± SEM of more than 3 independent experiments. ^*∗*^ *p* < 0.05 versus con group. ^#^ *p* < 0.05 versus TNF-*α* group.

Adhesion of BCECF-AM labeled HL-60 monocyte to HUVECs induced by TNF-*α* (50 ng/mL) was investigated. As shown in Figure 3, green fluorescent probes represent BCECF-AM labeled HL-60 cells. Monocyte adhesion was significantly increased by induction of TNF-*α* (50 ng/mL) for 6 h compared to the control group (^*∗*^ *p* < 0.05), whereas SMT (50 *μ*g/mL) pretreatment for 30 min significantly suppressed adhesion of HL-60 monocyte to HUVECs (^#^ *p* < 0.05).

### 3.4. Effect of SMT on TNF-*α* Induced ROS Production in HUVECs

**Figure 4:**
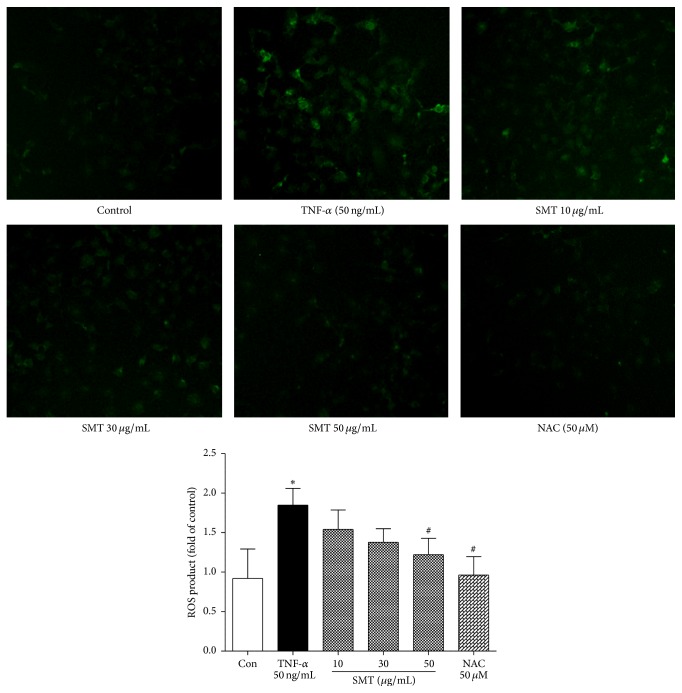
Effects of SMT on TNF-*α* induced intracellular ROS production in HUVECs. Cells were treated with TNF-*α* (50 ng/mL) for 6 hours in the absence or pretreatment of SMT (10, 30, and 50 *μ*g/mL) for 30 min and then treated with H_2_DCFDA. NAC (N-acetyl-L-cysteine) was used as ROS scavenger. Bar represents the mean ± SEM of more than 3 independent experiments. ^*∗*^ *p* < 0.05 versus con group. ^#^ *p* < 0.05 versus TNF-*α* group.

Intracellular production of ROS induced by TNF-*α* (50 ng/mL) was investigated. As shown in Figure 4, green fluorescent H_2_DCFDA represents generated ROS. TNF-*α* (50 ng/mL) induced HUVECs produced ROS compared to the unstimulated control group (^*∗*^ *p* < 0.05). However, SMT pretreatment inhibited ROS production against TNF-*α* induction and was significant in concentration of 50 *μ*g/mL (^#^ *p* < 0.05). Also, NAC (N-acetyl-L-cysteine), ROS scavenger, significantly blocked the production of ROS against TNF-*α* induction (^#^ *p* < 0.05).

### 3.5. Effect of SMT on TNF-*α* Induced NF-*κ*B Translocation in HUVECs

**Figure 5:**
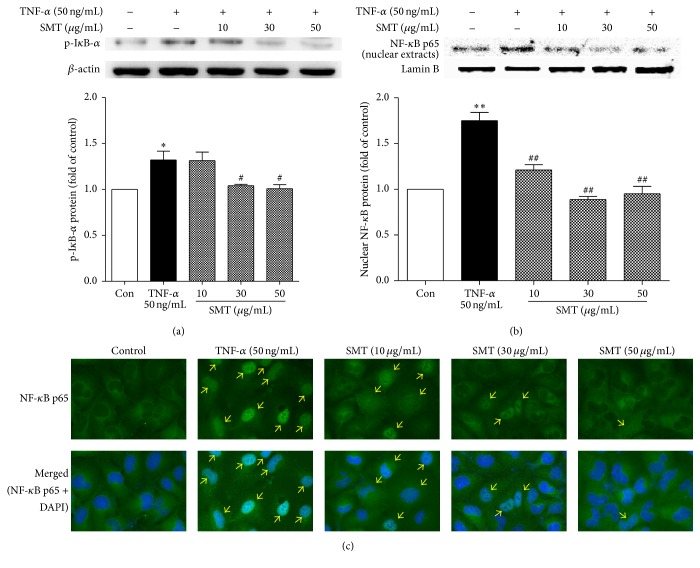
(a) Effects of SMT on TNF-*α* induced phosphorylation of I*κ*B-*α* in HUVECs. (b, c) Effects of SMT on TNF-*α* induced NF-*κ*B translocation in HUVECs. Cells were treated with TNF-*α* (50 ng/mL) for 1 hour in the absence or pretreatment of SMT (10, 30, and 50 *μ*g/mL) for 30 min. NF-*κ*B protein was detected by western blot and immunofluorescence. (Green: NF-*κ*B, blue: nucleus; magnification: 400x.) Bar represents the mean ± SEM of 3 independent experiments. ^*∗*^ *p* < 0.05 and ^*∗∗*^ *p* < 0.01 versus con group. ^#^ *p* < 0.05 and ^##^ *p* < 0.01 versus TNF-*α* group.

Nuclear and cytosol fraction extracts were isolated from HUVECs and western blot was performed to investigate effect of SMT on TNF-*α* induced phosphorylation of I*κ*B-*α* and NF-*κ*B translocation. As shown in Figure 5(a), phosphorylation of I*κ*B-*α* was significantly upregulated by induction of TNF-*α* (50 ng/mL) for 1 h (^*∗*^ *p* < 0.05); however, pretreatment of SMT for 30 min attenuated phosphorylation of I*κ*B-*α* and was significant in 30 and 50 *μ*g/mL (^#^ *p* < 0.05).

As shown in Figure 5(b) nuclear extract (NE) protein level of NF-*κ*B was significantly upregulated by TNF-*α* (50 ng/mL) induction compared to the control group (^*∗∗*^ *p* < 0.01) and this means NF-*κ*B translocated from the cytoplasm into the nucleus. Pretreatment of SMT for 30 min (10, 30, and 50 *μ*g/mL) significantly inhibited translocation of NF-*κ*B (^##^ *p* < 0.01). To visualize nuclear localization of NF-*κ*B, immunofluorescence was performed (Figure 5(c)). Green fluorescent NF-*κ*B is translocated into the nucleus by induction of TNF-*α* (50 ng/mL) for 1 h compared to the control group. SMT pretreatment for 30 min inhibited translocation of NF-*κ*B induced by TNF-*α* (50 ng/mL).

### 3.6. Effect of SMT on TNF-*α* Induced NF-*κ*B Activation in HUVECs

**Figure 6:**
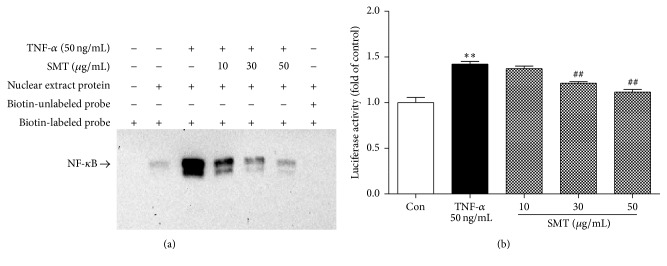
Effect of SMT on TNF-*α* induced NF-*κ*B activation in HUVECs. Cells were treated with TNF-*α* (50 ng/mL) for 1 hour in the absence or pretreatment of SMT (10, 30, and 50 *μ*g/mL) for 30 min and nuclear extracts were prepared to perform (a) electrophoretic mobility shift assay (EMSA) and (b) luciferase promoter assay. Bar represents the mean ± SEM of 3 independent experiments. ^*∗∗*^ *p* < 0.01 versus con group. ^##^ *p* < 0.01 versus TNF-*α* group.

Electrophoretic mobility shift assay (EMSA) and luciferase assay were performed to determine effect of SMT on TNF-*α* induced NF-*κ*B activation for further confirmation. In EMSA, as shown in Figure 6(a), NF-*κ*B-DNA binding complex shift is detected (Lanes 2–6) in nuclear protein added sample, but sample additionally added with a 20-fold excess of unlabeled oligonucleotide (Lane 7) did not show NF-*κ*B-DNA binding complex shift indicating that specific competitive reaction occurred and band was a NF-*κ*B specific shift. TNF-*α* (50 ng/mL) induction for 1 h (Lane 3) upregulated NF-*κ*B-DNA binding activity compared to the control group (Lane 2). SMT (10, 30, and 50 *μ*g/mL) pretreatment for 30 min (Lanes 4–6) inhibited NF-*κ*B-DNA binding activity against TNF-*α* induction in HUVECs.

As shown in Figure 6(b), luciferase promoter activity of TNF-*α* (50 ng/mL) treated cells was significantly increased compared to the control group (^*∗∗*^ *p* < 0.01). However, cells pretreated with SMT for 30 min inhibited luciferase activity against TNF-*α* (50 ng/mL) induction and were significant in concentration of 30 and 50 *μ*g/mL (^##^ *p* < 0.01).

### 3.7. Effect of SMT on HO-1 Expression and ROS Production in HUVECs

**Figure 7:**
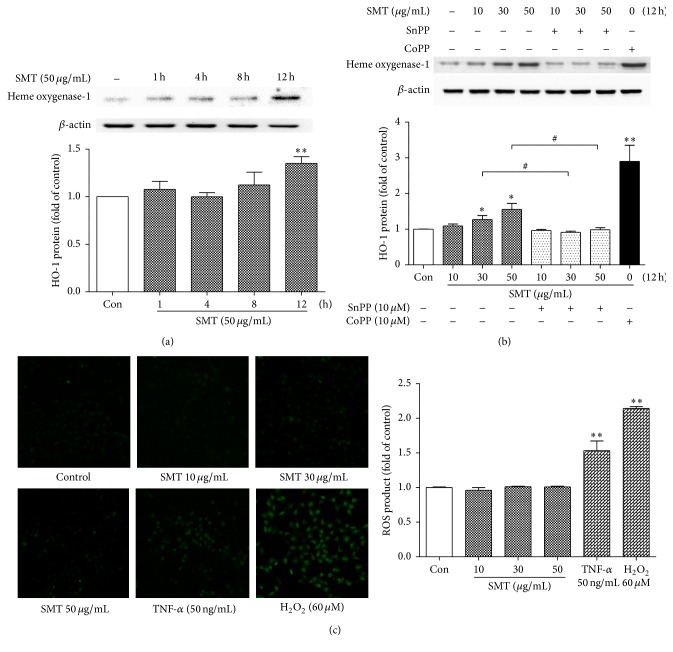
(a, b) Effects of SMT on heme oxygenase-1 induction in HUVECs. Cells were treated with SMT as indicated without TNF-*α*. (c) Effects of SMT on ROS production in HUVECs. Bar represents the mean ± SEM of 3 independent experiments. ^*∗*^ *p* < 0.05 and ^*∗∗*^ *p* < 0.01 versus con group. ^#^ *p* < 0.05 versus respectively indicated group.

To investigate whether SMT itself upregulates HO-1 protein expression in HUVECs, 50 *μ*g/mL of SMT is treated for 1~12 h. As shown in Figure 7(a), 12 h of SMT (50 *μ*g/mL) treatment significantly reached maximum HO-1 protein level (^*∗∗*^ *p* < 0.01). To confirm effect of SMT on HO-1 protein expression, SnPP (tin protoporphyrin, HO-1 inhibitor) and CoPP (cobalt protoporphyrin, HO-1 inducer) were used. As shown in Figure 7(b), 12 h of SMT treatment upregulated HO-1 expression and was significant in concentration of 30 and 50 *μ*g/mL (^*∗*^ *p* < 0.05); however, SnPP totally inhibited those effects (^#^ *p* < 0.05). CoPP dramatically upregulates HO-1 protein level (^*∗∗*^ *p* < 0.01). In addition, ROS was not produced by SMT treatment which means HO-1 was induced via ROS independent pathway (Figure 7(c)).

### 3.8. Effect of SMT on Nrf2 Translocation in HUVECs

**Figure 8:**
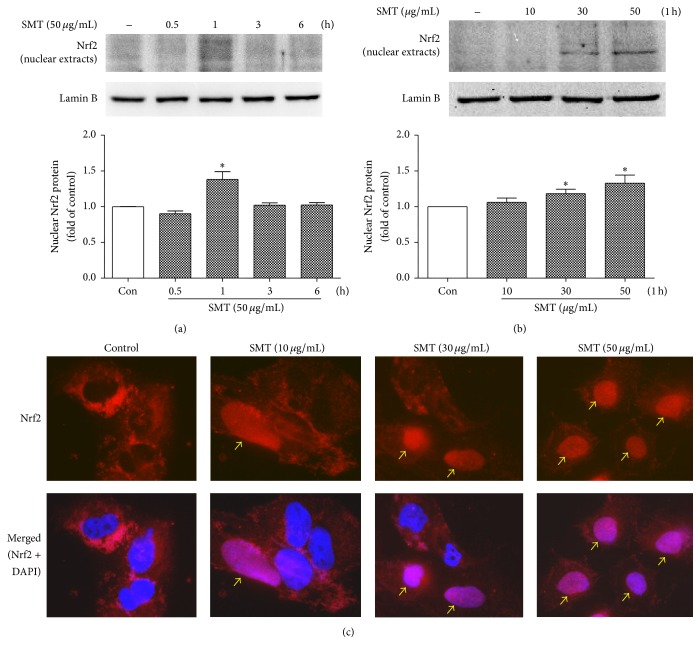
Effects of SMT on nuclear translocation of Nrf2 in HUVECs. Cells were incubated with SMT as indicated without TNF-*α*. Nrf2 was detected by (a, b) western blot and (c) immunofluorescence. (Red: Nrf2, blue: nucleus; magnification: 400x.) Bar represents the mean ± SEM of 3 independent experiments. ^*∗*^ *p* < 0.05 versus con group.

To further investigate HO-1/Nrf2 pathway, nuclear and cytosol fraction was isolated and western blot performed. SMT (50 *μ*g/mL) is treated for 0.5~6 h. As shown in Figure 8(a), 1 h of SMT (50 *μ*g/mL) treatment significantly reached maximum nuclear Nrf2 protein level (^*∗*^ *p* < 0.05). HO-1 induction by SMT treatment was significant in concentration of 30 and 50 *μ*g/mL (Figure 8(b)). Immunofluorescence was performed to visualize Nrf2 localization (Figure 8(c)). Red fluorescent Nrf2 was translocated into the nucleus by SMT treatment.

### 3.9. Effect of SMT on NO Synthesis

**Figure 9:**
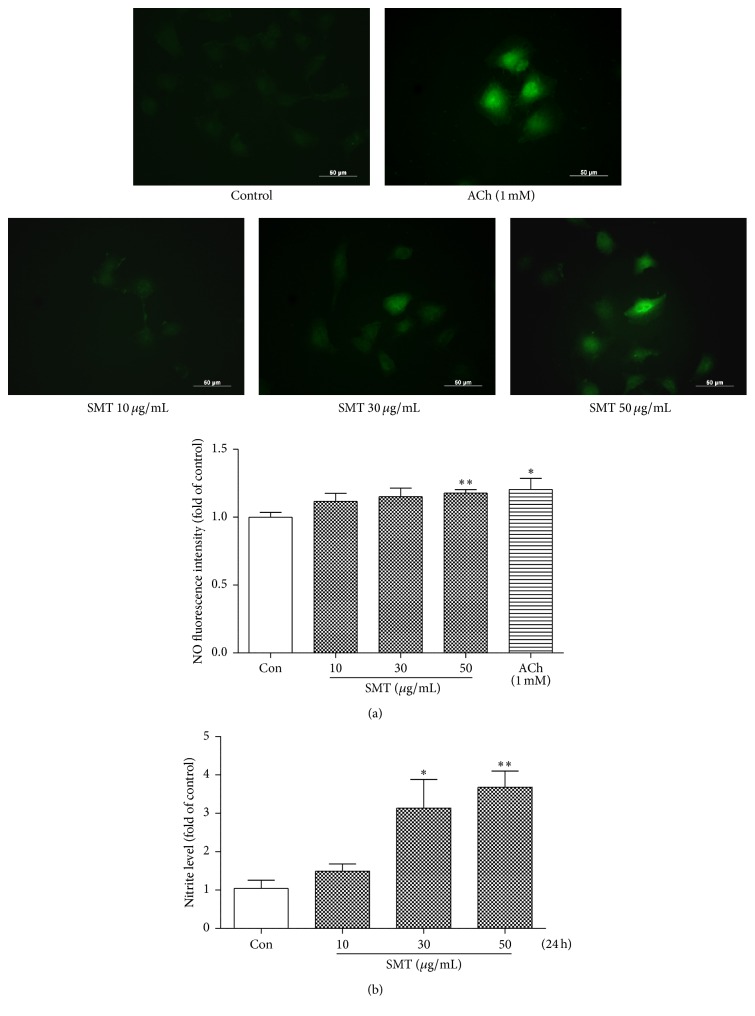
Effect of SMT on NO (nitric oxide) synthesis in HUVECs. (a) Cells were treated SMT or ACh for 30 minutes. DAF-FM diacetate was labeled as intracellular NO indicator. (400x magnification) ACh (acetylcholine) was used as positive control. (b) Supernatant of cell cultured medium was collected after 24 h of SMT treatment and performing Griess assay. ^*∗*^ *p* < 0.05 and ^*∗∗*^ *p* < 0.01 versus con group.

We measured intracellular NO and supernatant nitrite level to investigate NO synthesis ability of SMT on HUVECs. As shown in Figure 9(a), acetylcholine treatment resulted in intracellular NO synthesis and reacted with DAF-FM to fluoresce green. SMT treated HUVECs also synthesized NO and were significant in concentration of 50 *μ*g/mL (^*∗∗*^ *p* < 0.01). Nitrite level accumulated in supernatant of cultured medium measured by Griess assay was also increased by 24 h treatment of SMT (Figure 9(b)). The result showed a dose-dependent manner and was significant in 30 (^*∗*^ *p* < 0.01) and 50 (^*∗∗*^ *p* < 0.05) *μ*g/mL.

## 4. Discussion

This study showed that SMT suppressed expression of CAMs and monocyte adhesion via inhibition of ROS/NF-*κ*B activation induced by TNF-*α* and upregulated HO-1 and NO production in HUVECs. The major cause of atherosclerosis and other vascular diseases is chronic vascular inflammation and is initiated by proinflammatory cytokines such as TNF-*α*. TNF-*α* is produced from endothelial tissue resident immune cells, to upregulate the expression of adhesion molecules on endothelial cells [22]. Expression of CAMs such as ICAM-1, VCAM-1, and E-selectin mediates proinflammatory state and leads to formation of atheroma resulting in atherosclerosis [23, 24]. Pretreatment with SMT suppressed expression of ICAM-1, VCAM-1, and E-selectin and consequently attenuated adhesion of HL-60 monocytes induced by TNF-*α* in HUVECs. In expression of CAMs, NF-*κ*B activation is prerequisite and ROS have been implicated in all stages of atherosclerosis [10, 11] acting as second messenger [25]. ROS production results in phosphorylation of I-*κ*B-*α* and translocates NF-*κ*B into the nucleus. Therefore we further investigated ROS production and NF-*κ*B activation, upstream factor affecting expression of inflammatory genes including CAMs in HUVECs. I*κ*B-*α* (inhibitory *κ*B-*α*) is bound with NF-*κ*B and inhibits translocation of NF-*κ*B into the nucleus. Evaluating the concerned pathway, SMT pretreatment is found to suppress intracellular ROS production and phosphorylation of I*κ*B-*α*. These phenomena led to suppression of nuclear localization of NF-*κ*B and furthermore, results of EMSA and luciferase promoter assay showed NF-*κ*B-DNA binding activity was also suppressed by SMT pretreatment. SMT attenuated vascular inflammation by suppressing expression of CAMs, primarily resulting from inhibiting NF-*κ*B translocation and ROS production induced by TNF-*α* in HUVECs.

Recent studies suggest that HO-1 exerts vascular protective, antiatherogenic action and its expression in endothelial cells can attenuate atherosclerosis [26, 27].* In vivo* studies have shown that HO-1 knockout mice were vulnerable to chronic vascular inflammation [28, 29]. Nrf2 (nuclear factor-erythroid 2-related factor 2) is a transcription factor regulating several antioxidant effective genes and HO-1 is one of Nrf2-target genes [10, 11]. In the present study, we determined Nrf2/HO-1 level by treatment by SMT alone without TNF-*α*. The reason is that TNF-*α* acts as a negative stimulus increasing ROS production. As a result, negative feedback system of HUVECs could be activated to protect from damage and it might lead to HO-1 upregulation [30, 31]. There is possibility that HO-1 level solely affected by SMT could not be measured if both SMT and TNF-*α* treated. SMT treatment alone upregulated HO-1 induction in a dose-dependent manner and it resulted from Nrf2 nuclear localization. Furthermore, our data demonstrates that HO-1 production induced by SMT treatment in HUVECs did not result from ROS generation, suggesting SMT induced HO-1 via ROS independent pathway.

NO (nitric oxide) is a well-known vasodilator synthesized by eNOS (endothelial NOS) from L-arginine and eNOS knockout mice are known to represent endothelial dysfunction [32]. NO exerts vascular protective effects by regulating blood pressure, inhibiting platelet aggregation and leukocyte adhesion [33]. To investigate effects of SMT on NO synthesis in HUVECs, intracellular NO and NO_2_ (nitrite) secreted in medium are measured. DAF-FM, intracellular NO indicator, was preloaded with HUVECs in case of NO degradation. SMT treatment upregulated NO synthesis comparable to what acetylcholine did as a positive control and nitrite, an oxidative product of NO, was also found to increase in HUVECs treated with SMT for 24 h.

Relation between HO-1/Nrf2 and NO is controversial [34]. Heiss et al. reported that activation of Nrf2 led to increased intracellular NO level in primary human endothelial cells [35]. However, antioxidant effects of polyphenols result from NO mediated dissociation of Keap1-Nrf2 complex [36] and Pae et al. reported that NO can induce HO-1 particularly in endothelial cells [37] suggesting HO-1 as a biological target of NO. In the present study, whether Nrf2 translocation in HUVECs treated with SMT results from NO synthesis remains unclear. However, it is certain that, resultingly, numerous compounds in SMT led to induction of both intracellular NO and HO-1 in HUVECs and exhibited vascular protective effects.

Many researchers have studied with herbs and their compounds composing SMT are as follows: Angelica Gigantis Radix (*Angelica gigas *Nakai, root): its coumarin compounds such as decursin, decursinol angelate, and nodakenin upregulated HO-1 level in mouse vascular smooth muscle cells [38]; Cnidii Rhizoma (*Ligusticum officinale* Makino, rhizome): its phthalide derivatives such as ligustilide and senkyunolide were demonstrated to exert vasorelaxation action in rat isolated aorta [39]. So far which compound of SMT is responsible for vascular protective effect in HUVECs remains unclear and needs to be clarified in further study. Statins are a widely used drug for treating cardiovascular diseases inhibiting cholesterol synthesis [40]. Though statins can also pleiotropically attenuate inflammation or oxidative stress, adverse effects of statins are debatable [41] and some patients were reported to suffer from cognitive decline [42] and type 2 diabetes mellitus [43] due to statin medication. Therefore, investigating traditionally used herbal drugs such as SMT or “danshen dripping pill” [44] could be a complementary way to shed light on cardiovascular drug research.

## 5. Conclusion

SMT suppressed expression of CAMs via inhibition of ROS/NF-*κ*B activation induced by TNF-*α* and upregulated HO-1 and NO production in HUVECs. We suggest that four medicinal herbs of SMT, a traditionally used herbal formula, mutually cooperated with each other acting as a multitarget drug and might act as a promising vascular protective drug.

## Abbreviations

SMT: Samul-Tang
HUVECs: Human umbilical vein endothelial cells
TNF-*α*: Tumor necrosis factor-alpha
ICAM-1: Intracellular adhesion molecule-1
VCAM-1: Vascular cell adhesion molecule-1
E-selectin: Endothelial-selectin
ROS: Reactive oxygen species
NO: Nitric oxide
NF-*κ*B: Nuclear factor-kappa B
I*κ*B: Inhibitory kappa B
Nrf2: NF-E2-related factor 2
HO-1: Heme oxygenase-1.
